# Health-related quality of life in hypertensive patients with chronic kidney disease in low and middle-income countries

**DOI:** 10.1186/s12882-025-03957-z

**Published:** 2025-01-21

**Authors:** Wening Wulandari, Neily Zakiyah, Cherry Rahayu, Irma M. Puspitasari, Auliya A. Suwantika

**Affiliations:** 1 Department of Pharmacology and Clinical Pharmacy, Faculty of Pharmacy, Universitas Padjadjaran, Jalan Raya Bandung-Sumedang KM 21 , Jatinangor, 45363 Indonesia; 2 Centre of Excellence for Pharmaceutical Care Innovation, Universitas Padjadjaran, Jatinangor, Indonesia; 3 Hasan Sadikin Hospital, Bandung, Indonesia

**Keywords:** Low-income countries, Middle-income countries, Quality of life, Kidney disease, Hypertension

## Abstract

Hypertension and chronic kidney disease (CKD) are interconnected conditions that can significantly affect a person’s health-related quality of life (HRQoL). In low- and middle-income countries (LMICs), this disease burden is heightened due to limited health resources and socio-economic challenges. Based on the available literature, this narrative review aims to discuss the HRQoL of hypertensive patients with CKD in LMICs by identifying the current challenges and providing insights into the strategic potential to improve patient’s quality of life. This review reveals that the hypertensive population with CKD has a much lower HRQoL than the general population. Various factors, including physical limitations, comorbidities, psychological barriers, logistical challenges, and social support, can influence HRQoL. Limited access to health care, inadequate resources, and a lack of skilled personnel in LMICs further exacerbate these individual challenges. The economic impact of decreased work productivity and increased health costs adds to the disease burden. Improved health access, effective self-management strategies, and social support are needed to improve HRQoL in hypertensive patients with CKD.

**Supplementary Information:**

The online version contains supplementary material available at 10.1186/s12882-025-03957-z.

## Background

Hypertension is a major global health issue, responsible for an estimated 10.8 million deaths annually [1]. By 2019, it was estimated that 1.28 billion people worldwide were living with hypertension, yet only 30% of these individuals had their blood pressure adequately controlled [2, 3]. Uncontrolled hypertension is a key risk factor for the development of kidney disease, stroke, and cardiovascular disease (CVD) [4]. The relationship between hypertension and kidney disease is bidirectional; elevated blood pressure causes damage to kidney function, and the progression of kidney disease can worsen hypertension, creating a vicious cycle [5]. One of the primary mechanisms by which chronic hypertension contributes to chronic kidney disease (CKD) is the damage it causes to the renal vasculature, particularly through arteriosclerosis of the afferent arterioles, which reduces blood flow to the kidneys. Over time, this ischemic injury can result in glomerulosclerosis and progressive kidney damage [6–8]. Additionally, uncontrolled hypertension increases the risk of CVD, which can accelerate the progression of CKD to end-stage kidney disease (ESKD) [9–11].

CKD is associated with various complications affecting multiple organ systems, leading to a significant reduction in health-related quality of life (HRQoL) when compared to the general population [10, 12]. In hypertensive patients, symptoms such as headaches, chest pain, dizziness, and visual disturbances are common; however, these symptoms are not exclusive to hypertension and may also occur in individuals with other underlying health conditions [4]. CKD patients frequently experience symptoms such fatigue, weakness, nausea, vomiting, and decreased appetite, all of which further contribute to lower HRQoL. Even in the early stages of CKD, patients often experience comorbidities and require polypharmacy, including nephrotoxic drugs that may worsen kidney function [13]. The use of inappropriate or suboptimal treatments, such as medications that fail to adequately manage hypertension or that further impair kidney function also contributes to diminished HRQoL in hypertensive patients with CKD. Additionaly, side effects of treatment and family dynamics are crucial factors that influence HRQoL [14–16].

In low-and middle-income countries (LMICs), such as India, Nigeria, and Brazil, cardiovascular disease (CVD), including hypertension, accounts for about 10% of healthcare spending and necessitate lifelong treatment, particularly for patients with both hypertension and CKD. The healthcare systems in these countries are often under-resourced and face challenges such as limited access to medical treatment, which exacerbates the socio-economic burden and further decreases HRQoL [17]. The lack of advanced diagnostic tools, coupled with limited access to effective medications, makes it difficult to adequately manage hypertension and CKD, contributing to worsened health outcomes.

Polypharmacy is common among hypertensive patients with CKD, as these individuals often require a combination of antihypertensive agents, diuretics, statins, and nephroprotective drugs to manage their comorbidities. However, polypharmacy increases the risks of adverse drug reactions, drug-drug interactions, and nephrotoxicity, particularly in LMICs, where healthcare access and medication management are limited [4, 13–16]. This complexity, combined with the socioeconomic challenges faced by CKD patients, leads to a further decline in HRQoL.

The primary objective of this review is to evaluate the HRQoL of individuals with hypertension and CKD in LMICs by analyzing the available literature. We aim to highlight the challenges this population faces and propose strategies to improve their overall well-being and life satisfaction. This review seeks to contribute to ongoing efforts to reduce the burden of hypertension in CKD patients and improve health outcomes in LMICs by providing a comprehensive analysis of the current evidence and key challenges.

## Definition and context of LMICs

LMICs are classified based on their Gross National Income (GNI) per capita, as determined by the World Bank. LMICs have a GNI per capita ranging from $1,046 to $12,535, and they face distinct challenges that differentiate them from high-income countries (HICs). These challenges include limited healthcare infrastructure, lower government spending on health, and higher disease burdens, particularly from infectious diseases and non-communicable diseases such as diabetes and hypertension. The lack of sufficient medical resources, healthcare personnel, and equitable access to essential treatments exacerbates health disparities in LMICs [18]. As a result, health outcomes in these regions often reflect a combination of socio-economic factors, such as poverty, inadequate nutrition, and limited access to education, all of which influence the general well-being of populations.

The unique socio-economic and health-related characteristics of LMICs necessitate a tailored approach to public health and healthcare interventions. For instance, the burden of chronic diseases is increasing in many LMICs, but the healthcare systems remain underprepared to manage these conditions effectively. Furthermore, healthcare disparities between rural and urban populations, as well as significant inequalities in access to health services, further complicate efforts to improve health outcomes. In this context, understanding HRQoL in LMICs requires consideration of not only disease-specific factors but also the broader determinants of health, such as social support, community-based health initiatives, and cultural factors that influence health behaviors. Research focused on LMICs can provide valuable insights into how interventions need to be adapted to account for these structural challenges [19].

## The concept of health-related quality of life

**Fig. 1:**
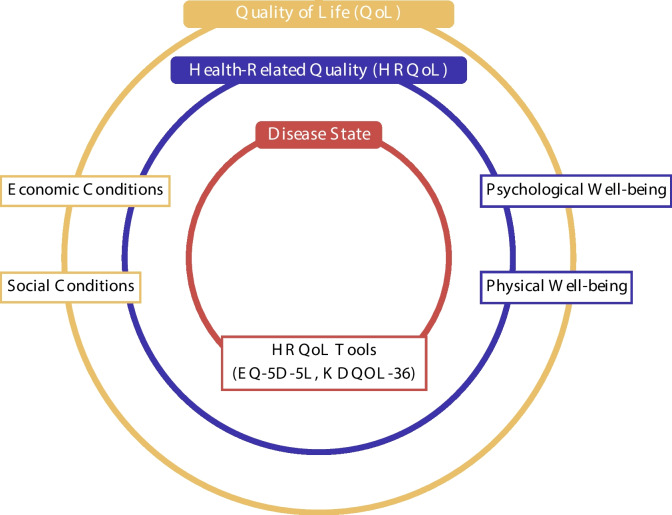
Illustration of the relationship between Quality of Life (QoL) and Health-Related Quality of Life (HRQoL). QoL, represented by the orange circle, includes a wide range of factors that contribute to a person’s overall well-being, such as economic and social conditions. These are non-health-related aspects of life that influence how satisfied individuals feel. HRQoL, shown in the blue circle, is a subset of QoL, focusing specifically on how a person’s health impacts their daily functioning and psychological well-being. It includes factors like physical and psychological well-being and is commonly measured using tools like the EQ-5D-5L and KDQOL-36. The innermost red circle highlights the impact of disease states on the body and well-being. This emphasizes that health conditions play a critical role in shaping an individual’s HRQoL, directly influencing their physical and emotional functioning. The use of distinct colors and circles helps distinguish between the broad scope of QoL and the more specific health-related focus of HRQoL

Quality of Life (QoL) encompasses a broad range of well-being aspects, including both health-related and non-health-related domains. While HRQoL focuses specifically on the impact of health conditions and disease states on an individual’s functional and psychological well-being. The notions of QoL and HRQoL are related, yet distinct from each other, as illustrated in Fig. 1. QoL includes all aspects of a person’s existence, such as economic and social conditions, and is defined as a conscious cognitive evaluation of life satisfaction [20, 21]. In contrast, HRQoL is narrower, focusing only on health-related factors [22]. It measures how well individuals function in daily life and their perceptions of their physical, psychological, and social health [23].

This distinction between QoL and HRQoL is particularly important in healthcare, especially in the management of chronic diseases. HRQoL measures help clinicians and researchers assess how health conditions affect daily functioning and emotional well-being, providing insights that go beyond mere symptom control to include a more holistic view of patient care [24]. Frequently utilized instruments to assess HRQoL include the Euro-Qol five-dimension five-level (EQ-5D-5L) and the Short Form-36 (SF-36) health surveys. The EQ-5D-5L evaluates mobility, self-care, usual activities, pain or discomfort, and anxiety or depression [25]. These instruments are widely used because they provide a comprehensive and standardized assessment, making it possible to compare different diseases and populations [22].

The choice of the most suitable HRQoL measurement instrument depends on the study’s objectives and the characteristics of the target population. Disease-specific instruments, such as the Kidney Disease Quality of Life (KDQOL-36), are often preferred for chronic kidney disease (CKD) patients, as they focus on health domains specific to kidney disease, such as the burden of illness and symptom [23] In contrast, generic instruments like EQ-5D-5L are ideal for comparing multiple health conditions and tracking broader population health trends. However, it is important to note that HRQoL instruments rely on self-reported data, which can vary across individuals due to differences in cultural, social, or educational backgrounds. This variation highlights the need for careful interpretation when assessing HRQoL across diverse populations [26].

Aslam et al. [27] conducted HRQoL measurements of hypertensive patients with comorbid CKD using the EQ-5D-5L instrument [28]. This tool rates health on five levels: “extreme problem”, “severe problem”, “moderate problem”, “mild problem”, and “no problem.” Furthermore, the EQ-5D-5L incorporates a visual analog scale (VAS) ranging from 0 to 100, where 0 represents the worst possible health and 100 the best. Due to its strong validity and reliability in chronic diseases, the EQ-5D-5L was chosen as the preferred tool for assessing the HRQoL of hypertensive patients with CKD [29]. The five-level scale also helps reduce the ceiling effect, making it more effective for HRQoL measurements [30–32].

## Impact of hypertension and CKD on health-related quality of life

Hypertensive patients with CKD often experience significant reductions in exercise capacity, which is typically measured through indicators such as maximal oxygen uptake (VO_2_ max) or treadmill tests [33, 34]. This decline in exercise capacity is attributed to the combined effects of hypertension and CKD, which contribute to muscle wasting, fatigue, and cardiovascular complications. Specifically, hypertension can cause vascular damage, while CKD leads to decreased renal function and electrolyte imbalances, both of which can impair physical performance. Studies show that patients with CKD and hypertension have a markedly reduced exercise capacity compared to healthy individuals, with reductions in peak VO_2_ max ranging from 15 to 40% in some populations [35].

Several factors, including poor physical functioning, multiple comorbidities, and both logistical and psychological challenges, further exacerbate this issue. Common symptoms like fatigue, shortness of breath, chronic pain, and limitations in physical function, as well as conditions such as cardiovascular disease and diabetes, contribute to reduced exercise capacity in these patients [31, 32, 36–40]. In LMICs, these challenges are amplified by limited access to healthcare resources, lower availability of structured exercise programs, and a lack of professional support. These factors further hinder patients’ ability to manage their conditions effectively and improve exercise capacity. In contrast, higher-income settings generally offer better healthcare infrastructure, access to rehabilitation programs, and professional support, resulting in more effective management of CKD-related complications and better HRQoL outcomes.

Additionally, psychological and cognitive barriers, such as fear of falling, feelings of being a burden, low motivation, depression, and lack of confidence, significantly hinder participation in physical activities. For example, in countries like India and Brazil, patients often experience social stigma related to their conditions, which worsens their reluctance to engage in physical exercise [35, 41]. Patients in LMICs also face logistical challenges, such as limited access to sports facilities, transportation issues, and difficulties scheduling exercise sessions due to work or family responsibilities [31, 36, 38, 39, 42, 43]. These barriers are particularly pronounced in LMICs, where healthcare systems are under-resourced and often lack the capacity to provide tailored exercise programs or adequate psychological support. Limited transportation options and financial constraints further compound these issues, making it difficult for patients to access even basic exercise or rehabilitation services. For instance, in Nigeria, transportation costs can prevent patients from attending rehabilitation programs, which are often located in urban centers [44]. Furthermore, non-dialysis CKD patients encounter additional structural barriers, such as insufficient access to exercise programs designed for their specific needs, lack of transportation to healthcare facilities, and inadequate guidance from healthcare providers, These challenges reduce opportunities for physical activity despite the well-established benefits of exercise for CKD management [31, 45].

The lack of peer support and professional support in patients with kidney failure significantly affects treatment adherence and confidence. Hemodialysis patients face additional challenges, such as insufficient training resources and limited access to endurance training equipment, which is often impractical due to size and cost [42, 43, 46]. While exercise specialists may be available, the responsibility for supporting physical activity often falls on medical staff, who may lack the motivation or expertise to guide patients effectively [36, 42, 47]. This lack of both peer and professional support is especially acute in LMICs, where healthcare providers are often overwhelmed, and patient education programs are not widespread, leaving many patients without the necessary guidance and training to manage their conditions optimally.

Hypertension, particularly when associated with CKD, has a profound impact on HRQoL. The condition often leads to decreased physical function, contributing to weakness, mobility issues, and increased risk of mortality. As glomerular filtration rate (GFR) declines, typically measured as estimated GFR (eGFR), it exacerbates muscle impairment and functional limitations, making it harder for patients to perform daily activities [48–50]. Additionally, the accumulation of intermuscular fat, further reduces muscle quality, leading to mobility issues, loss of independence, and heightened vulnerability to other health complications [51–53]. In LMICs, where access to advanced diagnostic tools and treatments may be limited, the progression of hypertension and CKD often goes undetected or inadequately managed, resulting in significant declines in HRQoL. The lack of effective management of these conditions contributes to a lower QoL, especially in terms of physical function, mental well-being, and social participation.

Emotional and psychological health is also a major concern for hypertensive patients with CKD, particularly those undergoing hemodialysis. These patients frequently report anxiety, depression, stress, and psychological distress [54–59]. They tend to have fewer coping strategies and experience more mental health challenges, requiring holistic care from a multidisciplinary team [60]. In LMICs, mental health support is often limited, and the stigma surrounding mental health issues may prevent individuals from seeking the help they need, further exacerbating their HRQoL. In contrast, patients in higher-income countries may have better access to mental health professionals, counseling, and social support programs.

Social support plays a crucial role in managing chronic diseases. Strong social networks are associated with better outcomes in conditions like cardiovascular disease, lung disease, dementia, cancer, diabetes, and CKD [61–66]. Among elderly individuals, inadequate social support is linked to poor HRQoL, higher risks of falls, cognitive decline, weakness, hospital admissions, and even death [67–70]. Social support also influences cognitive function, HRQoL, and frailty in older adults with CKD, making it an important factor in patient-centered care [71–73]. The role of social support is particularly crucial in LMICs, where family and community networks may be a primary source of support for patients with chronic illnesses. However, in urban settings or poorer regions, these networks may be stretched thin, limiting their effectiveness. In wealthier nations, organized social support services and community health programs are more accessible, improving HRQoL for individuals living with CKD. According to research by Dickens et al. [74], which examined older adults with non-dialysis-dependent CKD enrolled in the Chronic Renal Insufficiency Cohort (CRIC) Study, interventions to improve social support—such as house call initiatives, community engagement programs, exercise programs, self-care support groups, therapeutic counseling, and interactive discussion groups—may positively impact health-related quality of life, cognitive function, and frailty [75]. However, in LMICs, implementing such comprehensive interventions faces significant barriers, including limited resources, healthcare infrastructure, and trained personnel.

Pain or discomfort is one of the most significant issues for hypertensive patients with CKD, affecting 86.5% of patients, which in turn impacts their physical activity, mental health, and mobility. Effective management of these conditions can significantly improve the quality of life for these patients [27]. In LMICs, where pain management resources may be insufficient, and access to medications is often limited, the impact of pain on HRQoL can be more pronounced compared to higher-income countries, where pain management strategies and palliative care are more readily available.

## Healthcare access and utilization

In many LMICs, the annual per capita average health expenditure ranges between US$100 and US$400, significantly lower than the US$2000 observed in HICs. This disparity underscores the dual disease burden faced by LMICs due to the ongoing epidemiological transition [76]. These nations are grappling with the simultaneous challenges of communicable diseases, such as infections, alongside non-communicable diseases, including hypertension and CKD. Policymakers in LMICs must grapple with resource allocation decisions between combating communicable diseases that disproportionately affect the population under five years of age and addressing chronic non-communicable diseases like hypertension with CKD, which primarily impact adults and the elderly. This resource constraint often forces LMICs to prioritize immediate health crises, such as infectious outbreaks, over long-term management of chronic conditions like CKD, even though the latter is a growing issue.

Furthermore, the health systems in LMICs suffer from weakened infrastructure due to inadequate human resources, qualified workers, and facilities, leading to an unsustainable structure. Dysfunctional primary healthcare systems make it challenging to provide follow-up care for individuals with hypertension with CKD [77]. In contrast, higher-income countries benefit from stronger healthcare infrastructure, more accessible medical services, and a more robust healthcare workforce, making it easier to manage chronic conditions like hypertension and CKD.

Screening programs pose another bottleneck in LMICs, with the question of funding often arising. Limited data demonstrating the cost-effectiveness of community-based screening programs make it difficult for health policymakers in the region to make informed decisions about investing in hypertension with CKD screening initiatives. Policymakers should prioritize funding for screening programs, particularly for populations at high risk of CKD, because uncontrolled hypertension is the main factor in the occurrence of CKD [78]. In LMICs, limited funding for public health initiatives and a lack of robust healthcare data further complicate the implementation of large-scale screening programs. This is in stark contrast to higher-income countries, where such programs are often funded through public health initiatives and can reach a wider portion of the population.

The need for more relevant local data also hampers healthcare services in LMICs, making it challenging to plan effective prevention and treatment programs. The heterogeneity of different racial groups in LMICs necessitates localized data across various ethnic groups and languages. In addition, different blood pressure checks cause the risk of errors in the diagnosis of hypertension. According to the American Heart Association/American College of Cardiology (AHA/ACC) in 2017, the assessment of hypertension with CKD is carried out using two strategies, namely repeated visits to the doctor for pressure measurements or blood pressure monitoring outside the office [79]. In CKD, eGFR estimation equations may misclassify CKD due to differences in sensitivity across ethnic groups and CKD etiology. This issue is particularly relevant in LMICs, where access to accurate diagnostic tools may be limited, and where ethnic diversity may complicate the accurate assessment of kidney function. In contrast, HICs tend to have more standardized diagnostic practices and access to better tools for diagnosing CKD and hypertension.

The need for more skilled nephrology personnel presents a significant challenge in overseeing secondary and tertiary prevention programs. Moreover, the increasing number of diagnosed hypertensive patients with CKD exceeds the number of available workers. This shortage of specialized professionals not only limits the ability to provide timely and effective care but also contributes to increased workload and burnout among existing staff, further exacerbating the healthcare delivery gap in LMICs.

Globally, nearly 2 billion individuals do not have access to necessary medications, primarily in LMICs [80, 81]. In this context, the availability of statins, potassium-lowering agents, and steroids is crucial for managing the complications of CKD, particularly those that exacerbate hypertension. Statins help control dyslipidemia, which reduces cardiovascular risks in CKD patients, while potassium-lowering agents are vital for managing hyperkalemia, a common complication that worsens hypertension [82, 83]. Steroids, on the other hand, are used in cases of inflammatory or autoimmune kidney diseases to reduce inflammation and preserve kidney function, thereby assisting in blood pressure control and preventing further renal decline [84]. However, in many LMICs, these medications are not readily accessible due to high costs, insufficient healthcare infrastructure, and lack of government funding for chronic disease management. In HICs, these medications are more widely available, and treatment adherence is often better supported by healthcare systems.

Ensuring that these medications are readily accessible is critical for reducing the risk of hypertension-related kidney failure, particularly in high-risk individuals, given the high cost of renal replacement therapy (RRT) in many regions. Furthermore, it is essential for the effectiveness of programs aimed at controlling blood pressure and management of CKD [85, 86]. The high cost of RRT in LMICs, coupled with a lack of sufficient insurance coverage and government-funded healthcare programs, exacerbates the burden of CKD on individuals and families. In contrast, higher-income countries have better financial mechanisms, such as insurance systems or national healthcare coverage, that help mitigate the costs of RRT and ensure better access to necessary treatments.

## Work productivity and economic impact

Productivity refers to the efficiency with which inputs are transformed into valuable outputs for individuals, countries, or businesses. The presence of hypertension with comorbid CKD can have a significant impact on productivity. Research by Haalen et al. [87] primarily focused on the effects of anemia in CKD on quality of life and work productivity. While anemia is a common comorbidity in CKD, its impact on physical activity and productivity is distinct from the effects of hypertension. Further studies are needed to explore the specific impact of hypertension on work productivity in CKD patients, as reduced physical function in hypertensive CKD patients likely limits their ability to perform daily tasks effectively. At the same time, other factors, such as mental, emotional, and social issues can also contribute to decreased productivity. In countries like Australia, the overall public expenses and government support for kidney disease management reach $4.3 billion annually [88]. CKD and ESKD patients frequently experience higher rates of work absenteeism and reduced work productivity (“presenteeism”), compared to the overall population [87]. Additionally, hypertension accompanied by kidney problems is also associated with premature exit from the world of work. Nevertheless, the combined impact of work absenteeism reduced productivity, and early exit from the workforce due to comorbid kidney disease is rarely considered in health economics, and much of the published research on these aspects is outdated [89]. Reduced work productivity can have a significant impact on the economic status of hypertensive patients with CKD comorbidities, potentially increasing the poverty rate in LMICs and serving as a significant obstacle to the effective management of hypertension with CKD. The high prevalence of hypertension with CKD can result in large medical costs and a significant macroeconomic burden. Despite CKD disproportionately affecting individuals and families with limited resources, there are limited studies on the burden of hypertension with CKD, especially in LMICs in Asia [90–92].

## Strategies and interventions to improve health-related quality of life

Hypertension with CKD is often not prioritized on the World Health Organization (WHO) of non-communicable diseases, and only a few countries have clear policies or programs in place to prevent and treat CKD [93]. This lack of prioritization is particularly evident in LMICs, where limited healthcare resources, competing health priorities, and a focus on infectious diseases often leave chronic conditions under-addressed. Recognizing the complexities involved, effective management of hypertension in CKD patients is critical due to the bidirectional relationship between the two conditions. Uncontrolled hypertension not only worsens kidney damage but also increases the risk of cardiovascular complications, making blood pressure control essential for slowing CKD progression and preventing ESKD.

**Fig. 2:**
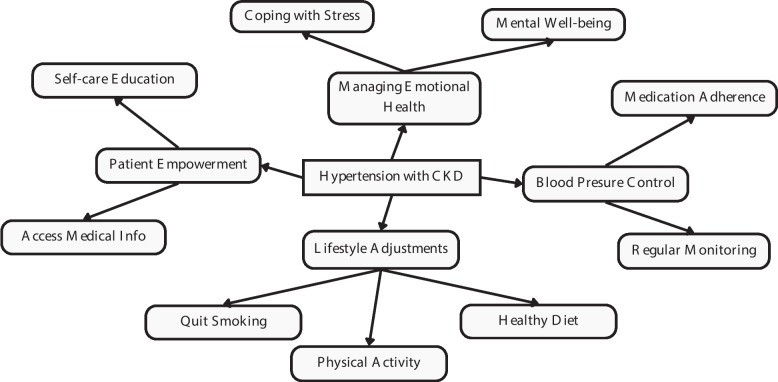
Comprehensive Self-Management Approaches for Hypertension with CKD

In LMICs, these challenges are further exacerbated by limited access to essential medications, lack of diagnostic tools for regular monitoring, and a shortage of skilled nephrology personnel. Infrastructure gaps, particularly in rural areas, and financial constraints also hinder the implementation of standardized treatment protocols. Given the complexities of managing hypertension in CKD patients, individualized therapeutic strategies are required to mitigate these risks in LMICs. Such strategies must consider resource limitations and focus on cost-effective, scalable interventions, such as patient education, lifestyle modifications, and improving access to affordable medications (see Fig. 2) [94].

Managing risk factors that can increase blood pressure and damage the kidneys is clinically important because it can prevent or minimize the likelihood of further blood pressure increases and kidney injury. Risk factors that can increase blood pressure include high sodium intake, low potassium intake, obesity, alcohol consumption, lack of physical activity, and unhealthy eating patterns. The Kidney Disease Improves Global Outcome (KDIGO) clinical practice guidelines advocate self-care as a model of CKD treatment, underscoring the integration of information, guidance, and education to promote self-management behaviors throughout all phases of CKD [95]. Similarly, the National Institute of Care and Excellence (NICE) guidelines, there is a recommendation to develop structures that foster self-care and empower individuals with CKD to make informed decisions about their health [96].

A promising approach to improving self-management and patient empowerment is the use of platforms like Renal Patient View or Patient Knows Best. These secure online systems provide patients with access to their medical information, including health and care data linked to medical records, allowing them to actively participate in managing their hypertension and CKD. Studies have shown that such platforms can improve patient engagement and self-management, leading to better hypertension control and CKD management outcomes [96, 97].

Self-management education aims to help patients lead active, productive lives while managing their hypertension with CKD. Key self-management behaviors for those living with hypertension and CKD include compliance with medication, health surveillance, symptom monitoring, and lifestyle adjustments (e.g., heightened physical activity and appropriate nutrition) to diminish cardiovascular risk factors, slow down CKD advancement, and enhance overall health [97]. Acquiring skills to manage the emotional ramifications of hypertension with hypertension comorbid CKD is vital for preserving mental well-being, nurturing a positive mindset, and adopting an optimistic approach to health and life. Participating in self-care practices may alleviate symptom burden, enhance quality of life, and potentially slow the progression of CKD due to hypertension [98, 99]. To facilitate self-management for individuals with CKD, it is widely advised that they possess an awareness of their diagnosis, partake in collaborative treatment decisions, access their medical information, and receive guidance on blood pressure regulation, physical activity, appropriate diet, medication administration, and quitting smoking [96, 100].

Engaging patients to actively participate in active involvement and commitment in their health and self-care endeavors is vital for enhancing overall health and HRQoL [101]. Understanding their chronic condition and its treatment is crucial for patient self-care and empowerment [102]. Possessing The essential competencies and awareness about their condition leads to better activation levels and increased engagement, thereby promoting improved self-management behaviors. Coexisting symptoms can negatively impact patient activation, everyday functioning, and self-care practices [103–106]. Moreover, Significant disease load and medication can hinder patient engagement and self-regulation, further exacerbating the already elevated incidence of depression and anxiety [107, 108]. Enhancing patient engagement aims to facilitate behavior alteration and enhance health results [101]. Positive improvements in activation have been associated with various positive changes in Self-care abilities for individuals with chronic conditions, such as participating in regular physical activity, coping with stress, following a suitable diet, and proficiently adhering to medication [109].

To effectively illustrate the various strategies and interventions aimed at improving health-related quality of life (HRQoL) among hypertensive patients with chronic kidney disease (CKD), we present a summary of relevant studies in Table 1. This table highlights the interventions tested, the populations involved, the outcomes measured, and the results obtained from these studies. By consolidating this information, we can better understand the effectiveness of different approaches, including educational programs, self-management strategies, and lifestyle modifications. This comprehensive overview will aid in identifying best practices and areas for further research, ultimately supporting the development of tailored interventions that address the unique challenges faced by this vulnerable population. Additional information about the included studies can be found in the supplementary file, Table S1.

**Table 1:** Summary of Interventions and Their Impact on Health-Related Quality of Life (HRQoL) in Hypertensive Patients with Chronic Kidney Disease (CKD)

Study	Intervention	Population	Outcome Measured	Result
Aslam et al. (2022) [27]	Educational program & peer support	Hypertensive CKD patients	HRQoL (EQ-5D-5L)	Significant improvement in mental health
Peng et al. (2019) [94, 97]	Self-management education program	CKD patients in early stages (stage 1–3)	Lifestyle modifications	Moderate improvement in self-care abilities
Teng et al. (2013) [100]	Targeted lifestyle modification	CKD patients	Physical activity and diet	Significant improvement in physical activity
Guerra et al. (2021) [54, 60]	Psychosomatic therapy for anxiety and depression	Hemodialysis patients with CKD	Mental health and HRQoL	No significant improvement in mental health
Fiaccadori et al. (2014) [36]	Physical exercise program	Hemodialysis patients with CKD	Physical function and HRQoL	Significant improvement in physical function

This table illustrates the diverse range of interventions evaluated in the context of hypertensive CKD, emphasizing both the successes and challenges of improving patient outcomes. It highlights the importance of targeted strategies tailored to the unique needs of this population, underscoring areas for future research and intervention development

## Future direction and research gaps

Further research is imperative to assess the effectiveness of interventions aimed at improving HRQoL in hypertensive patients with comorbid CKD in LMICs. Intervention studies can concentrate on strategies such as educational management, peer support networks, training programs, and social activity programs. Evaluating the impact of these interventions on HRQoL outcomes will provide valuable insights into practical approaches to enhance patient well-being. While longitudinal studies are essential to examine the long-term effects of hypertension and comorbid CKD on HRQoL in LMICs, we must acknowledge the challenge posed by high mortality rates in these populations. Patients who survive long enough for extended measurements tend to be the healthiest, introducing a potential selection bias. To address this limitation, future studies should incorporate strategies such as competing risks models, which account for mortality alongside HRQoL. Additionally, using proxy data from caregivers and shortening follow-up intervals can provide more accurate insights into the trajectory of HRQoL, even in patients at high risk of mortality.

Economic evaluation is another critical area that requires attention. Assessing the economic impact of hypertension and CKD comorbidities on individuals and the healthcare system in LMICs is essential. Such evaluations will enable policymakers to comprehend the costs of managing this condition and make informed decisions regarding resource allocation and financing for prevention and treatment programs. Understanding the economic burden of hypertension and CKD comorbidities can aid in the development of cost-effective strategies for managing and improving the quality of life for affected individuals.

Future research in the field of CKD management is critical to addressing the growing burden of hypertension and CKD, particularly in LMICs. Understanding the complex interplay of socioeconomic, cultural, and healthcare system factors in these regions is essential for developing effective interventions. Studies should focus on identifying key barriers to healthcare access, adherence to treatment regimens, and the role of patient education in improving outcomes. Additionally, research should explore the unique challenges faced by hypertensive patients with comorbid CKD in LMICs, including the availability of resources, healthcare infrastructure, and the impact of social determinants of health. By addressing these areas, future studies can provide valuable insights that inform evidence-based policies and tailored interventions to enhance HRQoL and ultimately improve patient outcomes in these underserved populations.

The review presents valuable insights into the HRQoL of hypertensive patients with comorbid CKD in LMICs. However, it is important to acknowledge certain limitations that may impact the generalizability and depth of the findings. One major limitation of this review is the lack of relevant local data in low- and middle-income countries (LMICs). Localized data are essential to accurately demonstrate the large and unmet medical care needs, especially in the management of hypertension and CKD. Without detailed data specific to the population and healthcare systems of LMICs, this description becomes overly generalized. Moreover, the absence of region-specific research underscores the need for further studies that focus on the epidemiology, healthcare delivery, and socioeconomic impacts of hypertension and CKD in LMICs. These data are critical for designing effective, evidence-based policies and interventions tailored to the unique challenges faced by these regions. Localized data are essential to accurately demonstrate the large and unmet medical care needs, especially in the management of hypertension and CKD. Without detailed data specific to the population and healthcare systems of LMICs, this description becomes overly generalized. Moreover, the absence of region-specific research underscores the need for further studies that focus on the epidemiology, healthcare delivery, and socioeconomic impacts of hypertension and CKD in LMICs. These data are critical for designing effective, evidence-based policies and interventions tailored to the unique challenges faced by these regions. The lack of longitudinal studies also restricts our ability to assess the long-term effects of hypertension and CKD on HRQoL over time. Additionally, the heterogeneity of ethnic groups in LMICs could influence disease presentation, risk factors, and cultural influences, potentially affecting HRQoL outcomes and making it challenging to devise universally applicable strategies. Furthermore, economic evaluations should make it easier for policymakers to prioritize and allocate resources effectively for hypertension and CKD management programs. Addressing these limitations through collaborative research efforts, longitudinal studies, culturally tailored interventions, and cost-effectiveness evaluations would strengthen the manuscript’s impact and contribute to more targeted and effective interventions for improving HRQoL in this vulnerable population.

Despite its limitations, this review offers important insights into the challenges faced by hypertensive patients with comorbid CKD in LMICs. It highlights the need for interventions to improve their HRQoL. Several key suggestions are proposed to address the identified issues and enhance HRQoL in this population. First and foremost, collaborative research efforts should be encouraged to foster international partnerships, enabling the collection of comprehensive and diverse data. This collaboration is critical for identifying context-specific challenges and solutions, particularly in LMICs, where resource constraints and population diversity require tailored approaches, and gather comprehensive and diverse data. Longitudinal studies are essential to assess the long-term effects of hypertension and CKD on HRQoL, enabling a better understanding of the trajectory of HRQoL over time and the factors influencing its changes. Moreover, economic evaluations are required to assess the economic impact of interventions and inform resource allocation decisions. Culturally tailored interventions are recommended to address the heterogeneity of ethnic groups and ensure that strategies resonate with specific populations. A multi-disciplinary approach is crucial, involving healthcare professionals, social workers, psychologists, and community support groups to provide comprehensive care and support. Capacity building and workforce training are needed to address the shortage of skilled nephrology personnel and enhance healthcare delivery. Public awareness and education campaigns can empower individuals to seek timely screening and adopt self-management practices. By implementing these suggestions, policymakers and researchers can effectively enhance HRQoL and alleviate the burden of hypertension with CKD in LMICs.

## Conclusion

Hypertensive patients with CKD in low- and middle-income countries face significant challenges that negatively impact their health-related quality of life (HRQoL). To improve HRQoL, it is essential to strengthen healthcare systems by enhancing access to screening, skilled professionals, and equitable treatments. Addressing polypharmacy through regular medication reviews, deprescribing when appropriate, and using digital tools to manage drug interactions can reduce adverse outcomes and empower patients to take control of their health, ultimately improving their well-being.

## Supplementary Information

Supplementary Material 1.

## Data Availability

No datasets were generated or analysed during the current study.

## Abbreviations

CKD: Chronic Kidney Disease
CVD: Cardiovascular Disease
ESKD: End Stage Kidney Disease
EQ-5D-5L: Euro-Qol Five-Dimension Five-Level
GFR: Glomerular Filtration Rate
HRQoL: Health-Related Quality of Life
HICs: High-income Countries
KDIGO: Kidney Disease Improves Global Outcome
LMICs: Low- and Middle-Income Countries
SF-36: Short-Form 36
QoL: Quality of Life
RRT: Renal Replacement Therapy
VAS: Visual Analog Scale
WHO: World Health Organization
