# Supplementary material for: Health-related quality of life in hypertensive patients with chronic kidney disease in low and middle-income countries
Source: BMC Nephrol. 2025 Jan 21;26:34. doi: 10.1186/s12882-025-03957-z (PMC11749374; doi:10.1186/s12882-025-03957-z)
Supplement: Supplementary file 1 — Supplementary Material 1. [file 12882_2025_3957_MOESM1_ESM.pdf]

S1. Information about the included studies

Study	Study design	Age group	Location	Sample size
Aslam et al. (2022)	Multicenter follow up study	≥18 years	Pakistan	517 patients
Peng et al. (2019)	Randomized controlled trial	≥18 years	China	2540 patients
Teng et al. (2013)	Randomized controlled trial	NA	Taiwan	160 patients
Guerra et al. (2021)	Observational study	18-70 years	Italy	113 patients
Fiaccadori et al. (2014)	Observational study	NA	Italy	104 patients

NA: not applicable.
